# Maximising impactful, locally relevant global mental health research conducted in low and middle income country settings: ethical considerations

**DOI:** 10.12688/wellcomeopenres.18269.2

**Published:** 2023-01-05

**Authors:** Clara Calia, Amit Chakrabarti, Emmanuel Sarabwe, Anna Chiumento

**Affiliations:** 1 Clinical Psychology, University of Edinburgh, Edinburgh, EH8 9AG, UK; 2 ICMR-Centre for Ageing and Mental Health, Division of Non-Communicable Diseases, Indian Council of Medical Research, Salt Lake, Kolkata, 700091, India; 3 Quality Assurance, Community Based Sociotherapy, Kigali, Rwanda; 4 Primary Care and Mental Health, University of Liverpool, Liverpool, L69 7ZA, UK

**Keywords:** Global Mental Health, Bioethics, Research ethics, Low and middle income countries, Community engagement, Impact, International partnerships

## Abstract

**Background:** Achieving ethical and meaningful mental health research in diverse global settings requires approaches to research design, conduct, and dissemination that prioritise a contextualised approach to impact and local relevance.

**Method:** Through three case studies presented at the 2021 Global Forum on Bioethics in Research meeting on the ethical issues arising in research with people with mental health conditions, we consider the nuances to achieving ethical and meaningful mental health research in three diverse settings. The case studies include research with refugees Rwanda and Uganda; a neurodevelopmental cohort study in a low resource setting in India, and research with Syrian refugees displaced across the Middle East.

**Results:** Key considerations highlighted across the case studies include how mental health is understood and experienced in diverse contexts to ensure respectful engagement with communities, and to inform the selection of contextually-appropriate and feasible research methods and tools to achieve meaningful data collection.  Related to this is a need to consider how communities understand and engage with research to avoid therapeutic misconception, exacerbating stigma, or creating undue inducement for research participation, whilst also ensuring meaningful benefit for research participation. Central to achieving these is the meaningful integration of the views and perspectives of local stakeholders to inform research design, conduct, and legacy. The case studies foreground the potential tensions between meeting local community needs through the implementation of an intervention, and attaining standards of scientific rigor in research design and methods; and between adherence to procedural ethical requirements such as ethical review and documenting informed consent, and ethical practice through attention to the needs of the local research team.

**Conclusions:** We conclude that engagement with how to achieve local relevance and social, practice, and academic impact offer productive ways for researchers to promote ethical research that prioritises values of solidarity, inclusion, and mutual respect.

## Disclaimer

The views expressed in this article are those of the author(s). Publication in Wellcome Open Research does not imply endorsement by Wellcome.

## Background

The ethical importance of achieving impactful and locally relevant mental health research in diverse global settings has long been recognised [see e.g. 1, 2], with the COVID-19 pandemic providing new impetus to this ^3– 6^. Mental health problems are a globally important public health issue in countries at different stages of economic development, and community-based mental health research is essential to understanding and responding to the growing burden of mental health problems in diverse global settings.

The breadth of considerations for impactful and locally relevant research range from research conceptualisation and prioritisation that is commensurate with local community understandings of mental health and expectations of mental health care; to methodological issues of contextually appropriate and feasible research design, methods, and measurement of mental health and related constructs. Frameworks for understanding mental health have been criticised for adopting ill-fitting models divorced from their sociocultural contexts ^7^. The mental health constructs adopted may not be contextually valid, and their focus on illness and disorder may exacerbate stigma, misinterpret local idioms of distress ^8^, and overemphasise the role of factors such as conflict-trauma at the expense of social determinants of mental health ^9, 10^. Research design can further exacerbate ethical tensions due to perceived prioritisation of care, or the rigidity of research designs [see this special issue for discussions from a previous GFRB on this topic: 11]. We also engage with the scope of what it means to discuss ‘impact’ – whether academic (e.g. peer review publications), the development of academic and practitioner collaborations and strengthening of local research capacity, or the social value of research or mental health care to participants and the wider community, including access to ancillary care and policy engagement and influence to embed services beyond limited research timelines.

Conducting impactful and locally relevant research also incorporates effective and respectful engagement with communities, and equitable partnerships with local government and non-governmental actors. Global research projects are built on principles of solidarity, justice, and fairness ^12^ embedded in a shared commitment to finding solutions to intractable, large scale and complex challenges. Ethics is a fundamental feature of contemporary global research, yet how ethics is conceptualised and enacted can vary by culture, institution, and discipline ^13, 14^. Furthermore, global research can include interactions between people with significant differences in power and voice, for example working with individuals who are highly vulnerable due to their socioeconomic status, ethnic or gender identity, physical and mental wellbeing, or being embedded in an inadequate legal and political protective environment. Global researchers must therefore be critically aware of their own practices and perspectives, as well as those of the individuals they collaborate or engage with ^15– 17^.

All of these considerations are reflected in the evolution of the scope and focus of the global mental health field which increasingly embraces an understanding of mental health and mental health care that are socially, politically, economically, and culturally embedded and constructed ^18– 21^. They raise underpinning considerations of community expectations of research(ers), the research project, and ancillary care, as well as for supporting local researchers engaging with the everyday complexities of research participants lives. They furthermore draw attention to the role of research funding – from the prioritisation of which topics get researched and using which research design and methods, to research timelines facilitating or restricting the opportunities for meaningful community engagement and partnership founded on common principles of solidarity ^22^.

We critically consider these issues through engagement with three case studies presented at the 2021 Global Forum on Bioethics in Research meeting on the ethical issues arising in research with people with mental health conditions. Through an examination of each case, we consider the nuances to achieving ethical and meaningful mental health research in three diverse settings, highlighting common strategies and approaches to achieving this.

## CASE 1: Ethical issues in research evaluating the implementation of Community-Based Sociotherapy in refugee settings in Rwanda and Uganda

### Background

The World Health Organization (WHO) indicates that the basis of effective mental health services is the prevention and management of common mental health problems at the community and primary health care level, with an emphasis on self-care ^23^. Community based Sociotherapy (hereafter Sociotherapy) has sought to adopt this model, with a history originating in the treatment of military casualties of the Second World War ^24^, and subsequently applied in the Netherlands to support the psychiatric treatment of refugees ^25^. When introduced in Rwanda in 2005, Sociotherapy was adapted to a community-based approach supporting Rwandans to respond to the consequences of the 1994 genocide against the Tutsi. Currently, Sociotherapy is implemented in communities, prisons, and refugee camps in Rwanda, the Democratic Republic of Congo (DRC), Liberia, and a refugee settlement in Uganda. In all of these settings, Sociotherapy as a group-based approach intends to support people whose lives have been disrupted by conflict and ongoing daily stressors. Its primary objectives include regaining and strengthening a sense of human dignity and psychosocial healing among participants.

Sociotherapy participants include people with a variety of psychosocial problems who do not need to have a mental health diagnosis. Sociotherapy groups are composed of fifteen people who live in the same neighbourhood and who meet weekly for three-hour sessions over 15 weeks. Each group is facilitated by two trained facilitators (Sociotherapists) from the same community as the participants, and group sessions follows six phases (safety, trust, care, respect, new life orientations and memory), with each session applying participatory methods to maintain participant engagement.

### Case study

Since November 2018, the Community Based Sociotherapy Adapted for Refugees (COSTAR) project - a collaboration between the University of Liverpool of UK, Makerere University of Uganda, and the University of Rwanda - has been conducting a cluster randomized controlled trial (cRCT) which aims to evaluate how Sociotherapy contributes to the reduction of depressive symptomatology in Congolese refugees living in settlement/camps in Uganda and Rwanda (See here for more information). COSTAR research participants are randomly recruited, after which they complete a pre-intervention survey and are randomly allocated to either the Sociotherapy intervention or the control arm (a group discussion around the Sustainable Development Goals and local community information). The ethical issues described in this case study draw upon the experiences of ES as the COSTAR coordinator of the Sociotherapy implementation, and his experience of conducting research in post-conflict settings.

1. Evaluating an adapted version of Sociotherapy according to a predesigned trial protocol: Sociotherapy is usually contextualised to the setting in which it is implemented. Recruiting participants involves Sociotherapists who know their community well identifying people likely to benefit from the approach. During the invitation process, the Sociotherapists lay the foundation for trust building between themselves and participants. Conversely, in COSTAR, Sociotherapy participants were randomly recruited by research assistants who were external to the community, and who conducted a formal pre-intervention survey as part of the recruitment process. After this, participants were randomly assigned to the Sociotherapy or the control arm; and a different research assistant linked the selected participants to Sociotherapists. This long process led by researcher assistants reduced trust between participants and Sociotherapists - a key component of the Sociotherapy process. This lengthy process may also have reduced the motivation of selected participants to engage in Sociotherapy groups, with potential negative impacts on the benefit they gained from the intervention as attendance in the COSTAR project was lower than sessions recruited via the usual approach adopted by Sociotherapy.
2. Responding to suicidal ideation: In the COSTAR screening process, people who were considered to have severe mental health problems, such as suicidal ideation, were to be excluded from further involvement and to be referred for specialist care. However, there were participants allocated to Sociotherapy who didn’t report having suicidal ideation in the pre-intervention and post-intervention surveys, but who during the Sociotherapy groups indicated that they had planned to commit suicide. These participants reported that Sociotherapy led them to abandon the idea of suicide, and therefore did not require referral to a specialist. In the usual practice of Sociotherapy, there is no formal screening to decide who participates in Sociotherapy, with the informal criterion being to exclude people manifesting noticeable severe mental health problem which cannot be managed in group sessions, ensuring the person is referred to a specialist for support. Due to the COSTAR cRCT recruitment process, participants with suicidal ideation may have been excluded, whilst our experience suggests that they may benefit from Sociotherapy.
3. Multiple layers of ethical and regulatory approvals: In the COSTAR project, all research adaptations had to be approved by the sponsor, the ethics committee of the University of Liverpool, and ethics committees in Uganda and Rwanda. The intervention was halted many times whilst the relevant approvals were obtained through this long (and expensive) process. These delays disrupted the intervention for both Sociotherapists and participants, which sometimes led to the de-motivation of participants and Sociotherapists, in turn leading to the cessation of both Sociotherapy and control arm sessions, further affecting the scientific validity of the cRCT.

### Critical reflections

Psychosocial interventions are promoted to heal wounds caused by violence and to restore social cohesion in the process of sustainable peace building ^26^. To test the effectiveness of these interventions, RCTs are considered gold standard, due to their procedures that aim to reduce researcher influence on data. However, because of the predetermined methodological standards of cRCTs, the COSTAR project imposed its methodological and ethical procedures upon the implementation of Sociotherapy. This approach meant that the intervention measured in the COSTAR project is different from the one usually implemented. It is possible that due to the adaptation of Sociotherapy to the demands of the cRCT, the intervention may be considered ineffective against the COSTAR specified outcome measures. However, for the reasons outlined, this research does not accurately capture or evaluate Sociotherapy as usually practiced, thus does not contribute to understanding its potential effectiveness in routine implementation. This raises ethical issues of the research designs, methods, tools, and procedures used to capture intervention effectiveness; and wider issues of participant inclusion and exclusion as research is conducted according to a random recruitment process and without delivery of the intervention to all participants, raising ethical issues of research benefits ^27^.

On a positive note, due to this experience the Sociotherapy team have learned about how to measure the effectiveness of Sociotherapy as it is usually implemented. In a subsequent research study currently being conducted the recruitment is led by Sociotherapists following the usual process of identifying people in the community who may benefit, and inviting them to participate without screening. After recruitment, participants are randomly allocated to either intervention arm or control arm, and all control arm participants will receive the intervention after completion of the research. In this way, the Sociotherapy team have been able to address the ethical tensions present in the previous COSTAR study, adopting a rigorous and ecologically valid research approach to provide insights into the efficacy of the Sociotherapy intervention.

A further recommendation arising from these ethical challenges is to localise procedural research ethics to avoid a long process of approvals. Allowing protocol adaptations to be approved by local ethical committee and/or having systems for local sponsorship and ethical review would reduce interruptions to intervention delivery whilst waiting for approvals, whilst maintaining scientific and ethical rigor ^28^.

## CASE 2: The need for the integration of health benefits as an ethical challenge in mental health research among low resource populations in India

### Background

The objective of this project was to develop a cohort (n=1526) of children of coal mine workers aged 6 to 23 years belonging to low socio-economic groups from rural and semi-urban areas in West Bengal, Bihar, Uttar Pradesh, as well as a tribal population. This project was part of a larger neurodevelopmental cohort “Consortium on Vulnerability to Externalizing Disorders and Addictions” (cVEDA, 2016 – 2020) carrying out a detailed assessment of behavioural, neuropsychological, neuroimaging and environmental exposures that can underlie externalising mental health problems ^29^.

### Case study

This case study examines the ethical implications of our experience that receiving tangible health benefits is perceived as the most important direct benefit which motivates participation in community-based mental health research in low resource settings in India. Our cohort comprised children of coal mine workers engaged in manual labour (skilled/semi-skilled/unskilled), as well as other children living in the mining neighbourhood. The cohort was drawn from a population of approximately 6,000 workers from 16 coal mines in the Asansol – Raniganj area of West Bengal, approximately 150km from the nearest metropolitan city of Kolkata. Male workers in this population are known to have a high prevalence of alcohol problems which is likely to affect vulnerability to mental health disorders among their offspring ^30^. Local community representative coordinators were engaged to assist research staff in participant recruitment. Following administration of informed consent, all participants underwent a detailed neuropsychological assessment and biological samples were collected. Neuroimaging was carried out in a sub-set of eligible participants who consented to this procedure, conducted in a government funded imaging centre in Kolkata, with travel arrangements and costs covered by the project. In our study we faced the following ethical challenges:

1. Conceptualisation of mental health as a barrier to participant recruitment: Our early realization was that for this population living in low socio-economic conditions the primary framework for understanding mental health is grounded in belief systems rather than a biomedical understanding. During our past and ongoing population-based studies a consistent observation has been a very low level of awareness about the existence of mental health problems, and mental health conditions are not perceived as a “disease”. This poses an ethical challenge in the implementation of research, as a result of the concept of mental health remaining obscure is that people do not come forward to participate in research on this topic. Through several periods of interactions with participants, parents, and the community as a whole, we were able to identify that there were efforts within the community to recognise mental health as a biomedical issue, and to encourage coming forward to seek help for this. We therefore felt that our model of mental distress was applicable to the local community understanding and ways of responding to mental health problems. Although it is desirable that the population has some understanding about mental health before initiation of the study, in our research a more pragmatic approach was adopted whereby we conveyed the concept of mental health during explanations about the study and the consent process. Therefore, we understood that alternate strategies are required to engage this population by foregrounding the relevance of the research to their current situation. In addition to explanations during participant information and consent processes, we also adopted wider engagement strategies. For example, considering the high prevalence of alcohol problems in this population we initiated a primary care screening and management of alcohol problems available to everyone in the community, and through participation in this screening we then introduced the study and encouraged individuals to bring their families to participate. This strategy was introduced in an effort to engage the local population by providing services to address a recognised problem in the community – alcohol dependence - and to assist in engaging more families to participate in the study.
2. Adequacy of, and collaboration with, local health services: The inadequacy of primary physical and mental health care remains a deterrent in implementing mental health studies in this population. During our project participants raised physical health issues and expected treatment advice and medication. We perceived that the reason for this could be the inadequacy of primary care service, which is unable to cater to the local population’s basic health needs. To address this, we collaborated with Physicians of Eastern Coalfields Ltd, a locally-based service provided free of charge, and referred participants with physical treatment needs for consultation. Second, we provided all participants with a complete hemogram (blood test) and routine urine report from biological samples collected for research purposes free of cost. Based on reports from these tests, we also tried to provide basic medicines (e.g. iron or folic acid, deworming tablets etc) at no cost through linking participants with government health programs, and for those who required further care, referrals to primary care physicians at the nearest care facility were made, where further management was provided. Anecdotal reports from participants indicate that this initiative was viewed positively, providing access to heath check-ups at a facility locally accessible to the community, and with the provision of basic medications free of cost.
3. Research benefits: We also felt that in low resource population settings the scientific benefits of research through the advancement of knowledge are intangible to the local community, and people are more concerned with receiving direct health benefits. Whilst some family members with children with obvious mental health issues (e.g., suicidality) were keen to participate, our experience was that participant’s primary motivation to engage in the study remained to receive physician consultation and medications.

### Critical reflections

Mental health issues related to personality, temperament and behaviour are, perhaps, perceived as social deviations rather than initiation of a disease process. To address this, in our study we explained to participants that such deviations may result in diagnosable mental health issues in the future, which need to be addressed at a very early stage to prevent more complex problems developing. One of the possible reasons for poorer understanding about mental health could be because this population struggle with receiving adequate physical health care.

We also encountered challenges due to the poor provision of mental health services at the primary care level. In our setting, participants with a provisional mental health diagnosis tend to seek Psychiatrist consultation. Considering the existing treatment gap for mental health services in India, it is difficult to arrange such services for this population at the primary care level. At the same time it is unethical for a participant with a provisional mental health diagnosis to remain uncared for. Therefore, in a country like India, to conduct ethical community-based mental health research requires the availability of mental health services at the primary and secondary care level. One of the main reasons people from low-income countries refuse to participate in health research is due to a lack of direct benefits from the study ^31^. We sought to address this by collaborating with local healthcare providers to ensure direct benefits to participants in the form of physical health tests and treatments, and to the wider local community in the form of an alcohol screening programme.

It is also important to emphasize that whilst minority population groups face a greater disease burden, they are known to be under-represented in health research ^32^. Ethically sound population-based research creates a strong evidence base to provide *locally* relevant and effective treatments for underprivileged populations. Our experience suggests that when participants are offered clear information to raise their awareness of mental health, and are provided physical and mental health services to meet their immediate needs, it creates a bond of trust between the researchers and the population. We recommend that researchers seek to build trust by understanding the health needs of the population in their socio-economic and cultural context, empathising with their needs, and acting to deliver meaningful direct research benefits. With this trust there is greater community advocacy about the study, facilitating spontaneous participation. This is an essential mechanism to generate a reliable mental health evidence base among underprivileged communities, which can assist in informing a more equitable allocation of health resources. 

This case study illustrates how mental health research in low resource settings is constrained by ethical challenges from the outset. When conducting community-based mental health research in low socio-economic populations of India, the application of ethical principles need to be contextualized to the socio-cultural context. Where the health needs in such communities are not met by currently available services, it is recommended that the researcher coordinates with local services to provide access for research participants and the wider community, thereby creating a bond of trust between researchers and the community, encouraging voluntary participation. Research participation shall result in awareness about mental health issues that may not currently be acknowledged at the community level, supporting both future mental health research and the mental health knowledge and understanding by the local community.

## CASE 3: Maximising meaningful and impactful mental health research for Syrian refugees during Covid-19 – co-creation, local relevance and ethical practice

### Background

The One Health FIELD Network brings together diverse, multidisciplinary expertise to increase food system resilience and support sustainable development in fragile and complex contexts. It draws together international and local partners, including: Syrian Academic Expertise for Agriculture and Food Security, various disciplinary schools from the University of Edinburgh, and the non-governmental organisation Cara (The Council for At-Risk Academics). It encompasses five pillars: Partnerships, Food Security and Safety, Gender Equality, Natural Resources, Livelihoods and Labour, and Health and Well Being.

*From the Field* was a research project launched by the Network to explore the impact of Covid-19 on the lives, livelihoods, and wellbeing of Syrians living in Lebanon, Iraqi Kurdistan, Jordan, Syria and Turkey using bespoke remote ethnographic approaches ^33^. Data was collected between April and September 2020, and explored the linkages between the psychosocial wellbeing of respondents and food security in a population facing enormous pressures and diminishing supports in their day-to-day lives. The University of Edinburgh and Cara identified and worked with local researchers to co-create and deploy questionnaire surveys using accessible technologies to 100 Syrian refugees in the region. These local research collaborators supported the co-creation of the survey, navigating Syrian idioms of distress and interrogating the proposed standardised measures to ensure these reflected local language and understandings, and reviewing and providing feedback on proposed recruitment procedures. They also offered qualitative feedback on their data collection experiences throughout the study.

### Case study

The central ethical question facing the project was how can we co-create an international research project with local mental health researchers/practitioners and displaced Syrians which is locally relevant and impactful? To support this, *From the Field* was underpinned by the Global Research Ethics Toolkit. Recognising the difficulties facing researchers operating within potentially constraining institutional environments, the Ethics Toolkit team captured the shared experiences of researchers around the world, which were then applied to develop tools which could support their research journeys. The toolkit was designed in two phases of collaborative work. The first involved a review of the literature ^34^, which informed the second stage of a set of consultations with researchers from around the world to create an online, open-access toolkit for the global research community. In all, the development of the toolkit was informed by more than 200 researchers from more than 30 countries, with over 60 disciplines represented ^34^. Rather than adopting a procedural ethics regulatory stance, this toolkit seeks to offer a flexible frame of reference to promote contextual ethical reflection and accountability within the research process and among research teams ^15^. The toolkit proposes two fundamental axes of reflective ethical analysis; firstly, iterative analysis throughout the ‘Research Journey’, and secondly, analysis based on the ‘4Ps’ model: Place, People, Principle and Precedent.

“Place” refers to the importance of considering the context in which the ethical dilemma is occurring and where the solutions must be applied. This raises a series of ethical issues associated with cultural differences between the place where the research is conducted and the different origins and customs of the research team. “People” refers to the different parties involved in the research investigation. Ethical conflicts are often associated with human relationships between participants and/or the research team. These include the researcher as an individual and his or her ethical conflicts, the research team biases and positionality, the participants, beneficiaries and stakeholders, and the establishment of ethical partnerships. “Principles” refers to the worldview and values that influence decision making, recognising that ethical challenges can be triggered by differences in principles that underpin and shape a research project, local context, and the worldviews of the people involved. Within these complex environments, the task of the researcher is to recognize these differences and then find pragmatic, values-informed solutions. “Precedent” refers to how history is shaping current and future challenges, including how we conduct research. Enabling ethical outcomes requires us to be alert to precedent so that we can discern when to draw on, and when to challenge, established ways of working.

**Figure 1.**
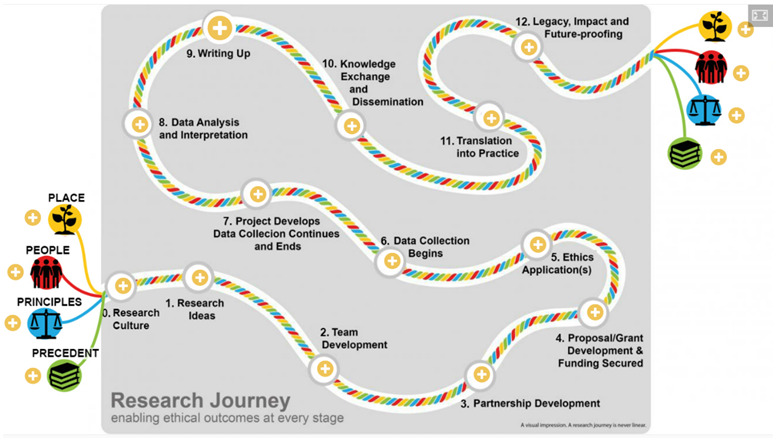
The Ethical Research Toolkit: Research Journey.

The Ethical toolkit also emphasizes ethical analysis as an iterative, reflective process relevant at all stages of the research journey. As such, the project developed a non-linear model of the stages of research, illustrating how the 4Ps are pertinent from *Stage 0. Research Culture* right through to the *12. Legacy, Impact and Future-proofing* ( Figure 1). Reflective analysis on the 4Ps throughout the research journey is achieved by following prompts relating to each of place, people, principle and precedent that aim to stimulate reflective discussion and collaborative engagement for each phase of the research journey. For example, when considering the ‘research idea’ stage prompts on ‘what harm could this research question bring?’ and ‘What benefits could be enabled?’ are actively discussed, surfacing potential ethical issues and supporting the refinement and selection of questions viewed as appropriate by all collaborators.

Shaped by the toolkit, the *From the Field* project developed a space for team members to work with respect for each other’s experiences and multidisciplinary knowledge to embed ethical reflections within the project’s collaborative practice. It provided a framework to guide team members to engage with a variety of complex practical and conceptual ethical challenges on an equal footing without assumptions or fear of judgement. To achieve this, *From the Field* through its collaboration with Cara placed Syrian and host community researchers at the centre of the design and implementation of the study. Working with Cara helped achieve the project’s goal to be meaningful and impactful by engaging a network of local experts who could support the research aims, while also enhancing protective links to at-risk academics. The collaborative discussions that followed resulted in recommendations to create a nascent network of expertise exploring long-term contingency planning for food security and health in Syria. It was imperative that the research questions guiding *From the Field* would be valuable to participants, contributing positively to the lives of those affected by the Syria crisis.

Collaborative discussions and reflections helped to identify the most appropriate tool for assessing mental wellbeing: The Short Warwick-Edinburgh Mental Well-Being Scale, S-WEMWBS ^35^, was selected for its cross-cultural validity in a wide variety of contexts, and for its translated version for Arabic speakers ^36^. Our local research collaborators included Syrian refugees or else speak the local dialect and have experience working with displaced Syrian communities, therefore benefitting from an emic understanding of participants’ cultural norms and circumstances. These local collaborators were engaged in a series of collective discussions supported by the Global Ethics Toolkit, and felt that this tool with its non-invasive, positively-worded orientation was suitable in this challenging humanitarian context, and it accounts for both dimensions of affect and functioning in order to capture changes in mood and the impact of daily stressors. The team reflected on the decision to identify measures which could do the best job at revealing the dimensions of psychosocial functioning over and above claims to making diagnostic categorisations. They also considered the extent to which this studies was contributing to identifying the elements of psychosocial functioning relevant to those affected by humanitarian crisis, with a view to enriching potentially simplistic descriptions of displaced persons as ‘vulnerable’ or ‘resilient’. It is important to note that this tool was one element of a wider project that explored subjective experiences of displacement and displacement related stressors through participatory methods involving food and music, as well as through qualitative interviews. In this way the S-WEMWBS tool was complemented by other methods of collecting, exploring, and disseminating the lived-through everyday dimensions to the displacement-related experiences of Syrians.

The toolkit’s ethical framework supported the team to ensure the psychological wellbeing of both displaced individuals and local mental health researchers. Participating in mental health research with displaced populations can itself have an impact on researcher mood and self-perception: a study can fulfil the expressed needs of displaced people to speak about issues affecting them, while local researchers may find themselves emotionally affected by participants’ responses. Research participants may be frustrated if their immediate needs are ignored in research. The sociocultural and linguistic background which made the local researchers more knowledgeable of the expressions of mental distress, also exposed them to stress. In humanitarian contexts, cultural counter transference can emerge between the researcher and the researched ^37^, and different types of empathy may manifest ^38^. To address this, *From the Field project* highlighted that mental health researchers require access to suitable training, clear referral pathways to respond to participants’ stated needs, protocols for situations of harm risk, and supportive supervision ^39^.

### Critical reflections

Conducting global research in conflict-affected contexts presents complex ethical challenges ^40^, including disrupting local power dynamics, the appropriateness of asking sensitive questions, and navigating risk for researchers and participants. The characteristics of ethical research, such as inclusivity, participation or accessibility, may themselves be contested. Such considerations have led some to argue for more nuanced guidelines and professional training, while others have emphasised a need to clarify feasibility, necessity and harm-benefit ratio ^41^.

In this study, we applied the Ethics Toolkit to support critical ethical reflections in team discussions throughout the research journey. Specifically, as a team, we reflected on the decision to identify measures which were felt to reveal the dimensions of psychosocial functioning relevant to displaced Syrians, over and above claims to diagnostic categorisations. We also considered the extent to which our study could contribute to identifying the elements of psychosocial functioning relevant to those affected by humanitarian crisis ^42^, for example by enriching a more nuanced understanding of potentially simplistic descriptions of displaced persons as ‘vulnerable’ or ‘resilient’.

Our experience in this study suggests that culturally-attuned, locally-driven mental health research is essential to a positive conceptualisation of mental health conditions, and is necessary if we are to understand the prevalence and presentation of common mental health problems in humanitarian settings. It also highlights that mental health research for displaced populations must ensure measures are fit-for-purpose in the sociocultural context in which they are used, and diagnostic tools and interventions should integrate an appreciation of the daily stressors people face and that act as social determinants of mental health. The ethical selection of research measures and tools includes a commitment to critically consider the necessity of asking sensitive questions, balancing potential harm to participants and researchers against epistemic knowledge gains.

If global researchers are to be successful in identifying and promoting solutions to the significant challenges the world currently faces, then they must be able to develop processes that allow stakeholders - including local researchers and host communities- to communicate around, and reflect on, the ethical dilemmas that arise. Developing strong and equitable partnerships, investing in the capacity of local mental health researchers, and supporting the development of ethical systems of researcher care so they can safely undertake research with those experiencing mental health distress, have been central ethical tenants in the design and conduct of this study.

## Discussion

The case studies presented highlight the breadth of considerations relevant to achieving impactful and locally relevant research. In our discussion, we consider core themes arising from these case studies and make recommendations for future practice.

The importance of wide and bi-directional stakeholder engagement and collaboration across high and low and middle income country (LMIC) partners, and with local research communities, and from research inception to conclusion, are recognised as essential for facilitating clear research expectations leading to mutual respect and trust ^13, 15– 17, 43^. In this process, stakeholders must be identified based on the research focus, which could include local mental and public health practitioners or organisations, policy makers, local community representatives including those with lived experience of mental health conditions, and other researchers or academic communities. To promote ethical engagement, these stakeholders need to have meaningful opportunities to shape and influence the selection of priority research questions, research design, conduct, and dissemination. Such inclusive collaboration can act to promote trust and sustain both community and researcher interest in mental health, avoiding tokenistic approaches to research ‘partnerships’.

Similarly, at the inception stage and throughout the research, and in collaboration with stakeholders, all facets of ‘impact’ should be identified. As the case studies demonstrate, this encompasses: social impact such as transforming local community understanding of, or attitudes towards, those with mental health conditions, and addressing research questions of priority to local communities; mental health services impact through the development and testing of mental health treatments or intervention implementation, including as a result of ancillary care or post-trial access to interventions; impact at the level of research infrastructure through the development of skills of local research team members or local mental health / public health practitioners, policy change or influence; and finally scientific impact through contributions to knowledge via traditional academic outputs (e.g. publications or presentations). It is also important to monitor and track impact during the research process, capturing both intended and unintended impacts arising from community, health system, and policy maker interactions, and recognising complex power dynamics that underpin these ^41^.

Our case studies also highlight the importance of recognising the competing demands upon mental health researchers who are motivated to address the needs of local communities, but whose operational context may be shaped by demands for bringing in grant income, publishing in academic journals, and career advancement ^44^. Compounding this is the often low-priority afforded to mental health in LMICs ^45^, which in turn shapes societal awareness and conceptualisations of mental health, and can make community engagement in research challenging. There are also tensions between the epistemic value of generating evidence to address inequities through identifying local needs to shape the provision of mental health care, and the constraints facing LMIC health care systems where physical and/or mental health care may be unavailable or of low quality ^46^. In such cases, the question is raised as to whether it is ethical to be identifying needs that cannot be met? Following on from this, it becomes important to consider to what extent researchers who are looking to address this inequity of health resources should be thinking about this in how they approach their research, for example, do they have an ethical responsibility to ensure that research is proposing solutions that are implementable in light of existing local resources and context? This would suggest that it may be unethical to conduct research that proposes solutions that are unaffordable, even if they contribute to a wider evidence-base. These tensions are embedded in a long history of global research and efforts to avoid ‘parachute’ research that involves conducting research on LMIC populations to generate evidence to be applied in other (most often HIC) contexts ^47^.

In addition, whilst the authors of this paper champion the role of procedural research ethics, there remains scope to enhance systems of procedural research ethics to better embrace contextualisation and collaborative and inclusive systems of review ^34, 40, 48^. The ethical review process is one opportunity for ensuring the local relevance of research to community needs and health system capacities, as well as assessing consideration such as direct benefit arising for the local community. We support calls for a fair system of procedural ethical review that seeks to meaningfully engage with the complexities of establishing equitable and inclusive international research collaborations working to conduct locally relevant research in diverse settings ^42, 49, 50^. Such a re-visioning of procedural research ethics is important for efforts to reform the ethical oversight of global mental health research ^51^, and to conceptualise procedural ethics as one phase of ethical engagement in the research journey ^15, 34^.

Our case studies have drawn attention to the key driver for achieving both meaningful and impactful mental health research is the mediating role of locally embedded researchers who bring knowledge of the research setting and context, and whose insight should be central to shaping how research is approached, including integrating novel and locally feasible research methodologies. This is particularly important when researching established interventions in LMIC contexts, taking steps to balance ecological validity of intervention implementation with the demands of scientific research standards; and in ensuring the relevance and cross-cultural / cross-language validity of research instruments or tools ^52^. Achieving this requires continued investment and support for the development of mental health researchers and practitioners in LMIC settings. Local teams equipped with the scientific and ethical knowledge to meaningfully inform, and lead, research through phases of conceptualisation, development, implementation, and impact for local communities and health systems are essential to achieving impactful and locally relevant research. Local researchers are also the ones embedded within settings and navigating the lived-through tensions inherent to conducting research, such as balancing social impact and scientific rigor, and championing local needs in international collaborations. As has been highlighted, achieving this requires attention to efforts to reform structural systems of research funding, ethical oversight mechanisms, publication and academic credit for research, and researcher career development ^53, 54^.

## Conclusion

Achieving impactful and locally relevant mental health research in diverse global settings is essential to informing mental health care that is grounded in norms of reciprocal solidarity and respect. Actively considering the dimensions of what ‘impact’ means in a given context and for a specific research project, and how research might ensure relevance for the local community, are two drivers of ethical mental health research. They seek to ensure that research is responsive to local community needs and respectful of local community ways of knowing, and of providing mental health support, balancing these against the scientific aim of advancing knowledge. Recognising pluriversal conceptualisations of mental health across cultural and linguistic contexts that are deeply embedded in diverse epistemologies, researchers must ensure meaningful stakeholder engagement to inform research prioritisation, design, implementation, and dissemination. Such steps promote equitable, respectful, and meaningful engagement to shape locally-relevant and impactful mental health evidence and services. The case studies discussed foreground the breadth of considerations inherent to achieving impactful and locally relevant research across structural systems of research funding, governance, and career development. We conclude that engagement with how to achieve local relevance and social, practice, and academic impact offer productive ways for researchers to promote ethical research that prioritises values of solidarity, inclusion, and mutual respect.

## Data Availability

No data are associated with this article.
