# Supplementary material for: Maximising impactful, locally relevant global mental health research conducted in low and middle income country settings: ethical considerations
Source: Wellcome Open Res. 2023 Jan 5;7:240. Originally published 2022 Sep 26. [Version 2] doi: 10.12688/wellcomeopenres.18269.2 (PMC10233314; doi:10.12688/wellcomeopenres.18269.2)
Supplement: Peer review history

# Peer review: Reviewer response for version 2

**DOI:** 10.21956/wellcomeopenres.20817.r56615

**Published:** 2023-05-31

**Authors:** Dónal O'Mathuna

**Affiliations:** 1 College of Nursing and Center for Bioethics, The Ohio State University, Columbus, OH, USA

Overall, this manuscript provides much useful information on an important topic: ethical issues in mental health research in challenging settings. The case studies are helpful in exploring more subtle and still significant ethical issues that arise in various stages of global health research. The format for discussing each of the cases was useful. The presentation of the research ethics toolkit with Case 3 was helpful in providing concrete suggestions and approaches to discussing planned research among the team and participant communities. This article makes an important contribution to the literature and, I believe, will be very useful in research ethics training courses and education.

 I do have a few areas where I give some suggestions for further revisions. These can be addressed with minor changes to the text, I think. In addition, I identified several typos. Please see the PDF file linked here for a copy of the manuscript where I highlighted these typos. Most of them have to do with verb tenses, but one in particular affects understanding what the author’s mean. The authors should carefully check the whole manuscript.

 In Case 2: I found it unclear who the participants were. They are introduced as “children of coal mine workers aged 6 to 23 years” (p. 5). Writing the ages closer to “workers” and not “children” suggests that the ages refer to the workers and these are the participants. Similarly, it’s unclear if it is the children or the mine workers who engage in manual labor (p. 5, top of second column). Then it states that the cohort came from 6,000 workers. Later references to the consent process suggests that adults are participants since young children would be involved in an assent process. Yet other parts suggest the participants are children. The confusion continues with reference to alcohol problems as its unclear whether the mental health concerns are for those with alcohol problems or the children of those dependent on alcohol. An explicit statement is needed to clearly identify the participants.

 The concern raised by an earlier reviewer about the presumed preference of a biomedical model of mental health issues remains. For example, the authors state: “Mental health issues related to personality, temperament and behaviour are, perhaps, perceived as social deviations rather than initiation of a disease process” (p. 6). It is unclear to me what is envisioned here, and I wondered if the authors include personality issues like those making relationships difficult, or temperaments like those of a very quiet person who appears withdrawn, or behavioral issues like anger outbursts. Communities might view expressions like this as social deviations, or as part of the range of social variation, or as behaviors which are socially unacceptable, but not necessarily a mental health problem or disease. In some communities such behaviors can be seen as moral, religious or spiritual issues, not issues of mental health or disease. The authors are aware of these issues given their use of an alcohol screening service to introduce their study. But in other places the article continues to suggest that getting people to see mental health through the disease lens is important. They claim that one of their ethical challenges was that participant communities did not perceive mental health conditions “as a ‘disease’” and that the concept of mental health remained “obscure” (p. 5). Note: the sentence starting with “This poses an ethical challenge…” contains a typo which makes it challenging to understand. Could it be that the ethical issue is that the researchers are insisting on a disease model for mental health when the local community has other understandings and does not agree or see the relevance of the researchers’ beliefs?

 The authors’ other points about addressing physical health and the lack of services for physical and mental health are very important and the case study is good at raising these as ethical issues. However, I would have liked to see the authors elaborate further on exactly what they found to be the ethical challenge in this research. Is it that basic health needs are not being met at the same time that expensive (I assume) neuroimaging and other tests are being conducted on participants? Or is it that holding a non-disease view of mental health is an ethical challenge? If so, in what way?

 Case 3. The explanation of the origins of the Global Research Ethics Toolkit is helpful. However, immediately after the hyperlinked Toolkit, I think a few words should be added to clarify that what follows is describing the original development of the Toolkit, not its adaptation to the Case 3 research. At first I was confused about why a local perspective was being developed by consulting researchers around the world. I would suggest adding something like: “the Ethics Toolkit was originally developed by the team capturing the shared experiences…” Another approach might be to put the material about the toolkit’s development and use into a separate box or side-bar since it isn’t part of the Case Study itself.

 On another note, the toolkit is named as the Global Research Ethics Toolkit, the Global Ethics Toolkit, and the Ethical Research Toolkit. At the linked site it is name Ethical Action in Global Research: A Toolkit. I think it would be best to stick to using one name consistently – or to remove the capitalization which suggests that this is a named instrument.

 In conclusion, I really liked this manuscript and commend the authors for it and their work in this important area. I plan to use the final article in my own teaching on research ethics.

Does the article adequately reference differing views and opinions?

Yes

Are all factual statements correct, and are statements and arguments made adequately supported by citations?

Yes

Is the Open Letter written in accessible language?

Yes

Where applicable, are recommendations and next steps explained clearly for others to follow?

Yes

Is the rationale for the Open Letter provided in sufficient detail?

Yes

Reviewer Expertise:

Disaster ethics and research ethics for disasters and humanitarian crises.

I confirm that I have read this submission and believe that I have an appropriate level of expertise to confirm that it is of an acceptable scientific standard.

# Peer review: Reviewer response for version 1

**DOI:** 10.21956/wellcomeopenres.20249.r52531

**Published:** 2022-10-24

**Authors:** Kaushalya Jayaweera

**Affiliations:** 1 Institute for Research and Development, Colombo, Sri Lanka

This article provides an overview of the complex ethical challenges involved in global mental health research. The authors discuss three case studies which demonstrate the challenges and barriers in engaging and involving participants from resource poor settings and conducting research. The critical reflections of each case study provide insights into how researchers in similar settings may manage the ethical challenges they face in their own research. With the availability of a myriad of publications on procedural ethics, articles such as these provide invaluable insights on overcoming potential ethical challenges and barriers involved in field research.

Does the article adequately reference differing views and opinions?

Yes

Are all factual statements correct, and are statements and arguments made adequately supported by citations?

Yes

Is the Open Letter written in accessible language?

Yes

Where applicable, are recommendations and next steps explained clearly for others to follow?

Yes

Is the rationale for the Open Letter provided in sufficient detail?

Yes

Reviewer Expertise:

Epidemiology, ethics, mental health, non-communicable diseases, public patient involvement and engagement

I confirm that I have read this submission and believe that I have an appropriate level of expertise to confirm that it is of an acceptable scientific standard.

# Peer review: Reviewer response for version 1

**DOI:** 10.21956/wellcomeopenres.20249.r52530

**Published:** 2022-10-10

**Authors:** Kenneth E. Miller

**Affiliations:** 1 Department of Educational and Counselling Psychology, and Special Education, The University of British Columbia, Vancouver, BC, Canada

The authors are to be commended for addressing a critical set of ethical issues related to conducting research in low-income communities and populations with different cultural backgrounds than those of the lead researchers. The title doesn’t accurately reflect this focus, and I’d suggest modifying it (it currently just speaks about locally relevant research, but the examples are all of global health/mental health researchers working in low-income settings, and their discussion seems to focus on research in such settings). 

 A strength of the paper is the use of case studies to illustrate and engage with key ethical issues. This approach can help authors move beyond abstract, intellectual discussion by grounding their arguments in concrete examples. The first case study is an excellent example of this approach, illustrating clearly the trade-off in validity when imposing an overly rigid RCT design to evaluate a community-based psychosocial intervention. The solutions that were developed in the subsequent study of sociotherapy nicely illustrate how good science can evolve out of innovative thinking coupled with a genuine dialogue between researchers and local practitioners and other knowledgeable stakeholders.

 Unfortunately, I found the subsequent two case studies less compelling. Case #2 seems to regard the disease model of mental distress/illness as the true model, and a problem in the local community was their lack of awareness of this correct model. In fact, many mental health researchers, including many trained in high income settings, also see mental health problems as reflecting social determinants rather ascribing to a disease model. The researchers acknowledge that some of the resistance to the research project stemmed from difficulties among participants in seeing the relevance of a basic science study in a context of considerable deprivation. It seems like their solution was basically to encourage participation through the provision of blood tests and urine samples which were then used to make medical referrals, and through screening people for alcohol problems. It’s unclear to me how the relevance of the research was ever truly conveyed and agreed upon by the target community—a key argument made in the paper, or whether and how the alcohol screenings were used to link people to treatment services (why screen without treating?). Also, the authors say they provided meds, but they did not; they provided test results and referrals, which isn’t the same as providing meds. I would modify the wording to make this more accurate. Did people use the referrals and get the meds or other services they needed? Did the authors follow up on this?

 Case #3 is perhaps the least concrete of the three. I had trouble understanding what The Global Ethics Research Toolkit involves—and while I was able to look it up online, ideally a reader should be able to get a clearer sense from the paper itself. I understand that the team followed the concept of “reflective ethical analysis”, and the 4Ps model, but I don’t actually understand what this meant in practice. I was surprised that the short form of the WEMWBS was selected due to its “validity with Arabic speakers”. Arabic speakers comprise an extraordinarily large set of ethnically and nationally diverse groups, and the validity of the WEMWBS has only been established for a very small number of them. Syrians are Arabic speaking, but one wouldn’t want to assume validity of the measure because it had been validated on another Arabic speaking population. If the authors meant that it was selected because of its validity with Syrians, then this should be clearly stated and a reference included. I was also a bit surprised by the choice of the measure, which I like and have used, in light of the authors’ discussion of the importance of understanding Syrian idioms of distress and capturing the impact of displacement on Syrians. The short form of the WEMWBS is good, but doesn’t capture any Syrian idioms of distress, nor does it assess anything close to a representative sample of impacts of displacement and displacement-related stressors. The authors note, accurately and importantly, that researchers “must ensure measures…are appropriate to the sociocultural context in which they are used, and diagnostic tools and interventions should integrate an appreciation of the daily stressors people face.” Absolutely true, but it’s unclear to me from the case report to what extent this group of researchers actually did these things. Perhaps the report could spell that out more clearly.  Likewise, Case #3 suggests the importance of a host of ethical considerations, yet doesn’t illustrate how these were enacted or addressed in the study being presented (e.g., considering all facets of impact, capturing both unintended and intended impacts, etc.). It’s fine to create a list of ethical considerations, but I think the power of case studies is to illustrate concretely how specific issues were encountered and addressed, as was done so well in Case 1.

 The Discussion is among the richest and most compelling sections of the paper. Some critical points are raised, none of which have yet been adequately answered in the literature, and the authors are to be commended for raising them here. Chief among these is the thorny question of whether it is ethical to propose solutions based on one’s research that are not feasible in the real-world context where the research has been conducted.  

 Two small points in the Discussion: if the authors wish to refer to ethical oversight as colonialist, they should make the case why this is so and not simply state it (as they do when they speak of “decolonializing ethical oversight”). Secondly, I would urge the authors to use accessible language; I have no idea what *pluriversality* is, and the citation next to the term does not make me want to go hunt down the meaning of the word.

Does the article adequately reference differing views and opinions?

Partly

Are all factual statements correct, and are statements and arguments made adequately supported by citations?

Partly

Is the Open Letter written in accessible language?

Partly

Where applicable, are recommendations and next steps explained clearly for others to follow?

Partly

Is the rationale for the Open Letter provided in sufficient detail?

Yes

Reviewer Expertise:

Mental health, armed conflict, and displacement

I confirm that I have read this submission and believe that I have an appropriate level of expertise to state that I do not consider it to be of an acceptable scientific standard, for reasons outlined above.

# Response

**Published:** 2022-12-20

**Authors:** Anna Chiumento

**Affiliations:** University of Edinburgh, UK

We are grateful to both reviewers for their thoughtful comments and reflections on our manuscript. Both reviewers note the contribution this article makes to discussions of research ethics when conducting global mental health research in low and middle income countries.  The reviewers both appreciate the foregrounding of the lived-through experiences of conducting research that illuminate a range of ethical challenges through the presentation and critical engagement with case studies.  Finally, both reviewers recognised the depth of the discussion in seeking to draw together critical reflections arising across the three case studies, and the importance of raising some challenging questions that the field have yet to fully address.

 Reviewer 1 provided constructive feedback in relation to the case studies and discussion, which we have engaged with.  Here we address each of his points in turn:

Clarity of the title: Thanks for highlighting the lack of clarity in the title, which we have amended to “Maximising impactful, locally relevant global mental health research conducted in low and middle income settings: ethical considerations” to further specify the article focus to readers.

Case study 2: Thank you for highlighting the important issue of the relevance of a biomedical approach to addressing mental health problems in case study 2.  In this study, one of the successes of the research was that through several periods of interaction with parents, peers and the community as a whole, we were able to identify pre-existing community efforts to recognise mental health as a biomedical issue, and to encourage seeking help for this. We therefore felt that our model of mental distress was applicable to the local community understanding and ways of responding to mental health problems. We have amended the manuscript text to clarify this point.  We would also like to highlight that we made efforts to convey the relevance of our research and the need to identify mental health issues at an early age for improved developmental outcomes. We organized awareness sessions in the community in different neighbourhoods, spread the messages in local schools, organized small group meetings among peers in the community, and discussed with officials in the mining areas; so that the community could be sensitized at different levels. Having identified this, we then sought the services of a qualified psychiatrist on a periodic basis who visited the local primary care facility to assess, review, and treat those experiencing mental health difficulties.  Any participants with possible mental health issues identified during study assessments were evaluated and strategies for management, including referral, were provided. On similar lines, coal mine workers who exhibited a hazardous and/or dependent pattern of alcohol use following screening were assessed by the visiting psychiatrist for management and further referral, if necessary, was provided (this is included in the existing text where we state: “we initiated a primary care screening and management of alcohol problems available to everyone in the community, …). This screening and management was available to everyone, regardless of engagement in the research, seeking to ensure that these services did not act as undue inducement for research participation. We would also like to clarify that in this study the research team did provide medication through Government funded public health programmes which participants who required these were registered with, depending on their test results (e.g., providing iron and folic acid for iron deficiency anaemia, albendazole for deworming etc). Participants who required more systematic evaluations were referred to primary care physicians at the nearest care facility, and further management was provided by them. Many participants and their parents reported back to our field staff regarding the outcome of their primary care visits following referrals, with this initiative receiving a positive response as the participants were able to access the necessary heath check-ups; basic medications were provided free of cost through the primary care facility; and as the facility was located in the same neighbourhood participants did not have problems regarding accessibility.  Again, further clarifications have been made to the main text regarding these points.

Case study 3: Thank you for highlighting the need for a more comprehensive description of the Ethical Research toolkit, which is a recommendation we agree with.  A paragraph has been added to the paper that describes the toolkits development through a consultative and co-construction process, provides references for the reader to explore further, and offers a full explanation of the toolkit model and process for supporting ethical research practice, including description of the 4Ps and how this is applied to facilitate reflective ethical analysis. Thank you for foregrounding consideration of the relevance of the Arabic translation of the sWEMWBS for Syrian participants.  As highlighted in the text, the scale was first translated to Arabic by Zayed (2021) following the translation and adaptation recommendations of the International Test Commission (internal consistency 0.91, reliability using test–retest method was 0.79). A fully validated version is not available.  We have clarified this in the text, where the language of “validity” has been replaced with “translated”. As the reviewer notes, the WEMWBS has been used extensively in cross-cultural mental health research, and has demonstrated good psychometric properties in diverse populations. The full WEMWBS has an official Arabic translation which has been used with Syrian refugees (for instance in a Lebanon RCT by Miller et al, 2020.  In our study, the sWEMWBS measure was selected by our Syrian colleagues because of their views that it was felt to be the most appropriate to their sociocultural context, and avoided clinically pathologising a population exposed to humanitarian crisis. To support this discussion, we applied the Global Ethics Toolkit (specifically sections on Research idea - What harm could this research question bring? What benefits could be enabled?; and Data Collection - Do our data collection processes present any potential risk or discomfort for the participants or end users, or for researchers or partners?), to stimulate discussion with our Syrian colleagues about the most appropriate measures to the socio-cultural context. We met regularly online before, during and after the data collection. Because our data collectors are either themselves Syrian refugees or speak the local dialect and have experience working with displaced Syrian communities, our network benefits from an emic understanding of participants’ cultural norms and circumstances, enabling close consideration of the appropriateness of this tool, and supporting the conduct of our research with due care and sensitivity. It is also important to note that the administration of this tool was embedded in a larger research project which also included Syrian Humming project https://syrianhummingproject.com/ and the SyrianFoodFuture https://syrianhummingproject.com/. Participants were able to describe their difficulties, but also their resilience, through their involvement in different ways: food, music, interviews, and the publication of a graphic novel *“May God Bless the Hand that Works”: Stories from Displaced Syrian Farmworkers during the COVID-19 Pandemic *( https://www.onehealthfieldnetwork.org/may-god-bless-the-hand-that-works).  In these ways local subjective everyday experiences of displacement were being captured, providing rich accounts to complement the structured data obtained through the S-WEMWBS. This information has been added to the article to situate the use of this tool.

Discussion: Many thanks to Reviewer 1 for an overall positive assessment of the discussion.  We hope that by raising the ‘thorny’ issues these will be further engaged with by those working in the field, and although we accept there are no easy answers, our aim is to foreground awareness of the ethical choices being made at each stage of the research journey.  We appreciate the two points in relation to the clarity of language, and have firstly removed reference to pluriversality, which we agree with the reviewer is not clear terminology and would be confusing, and unnecessary to the point being made, to start defining in the discussion.  And secondly have amended the declaratory sentence about ethical review as colonialist, which we agree is a point of ongoing debate, to state: *“As has been highlighted, achieving this furthermore requires attention to efforts to reform structural systems of research funding, ethical oversight mechanisms, publication and academic credit for research, and researcher career development”.* We hope that these responses and the ammendments to the article satisfactorily address the reviewers feedback.
